# Low-Grade B-cell Lymphoma in Primary Sjögren’s Syndrome: A Case Report

**DOI:** 10.7759/cureus.99140

**Published:** 2025-12-13

**Authors:** Thanda Aung, Giovanni Botten, Anamaria Munteanu, Bita V Naini, Anthony Pastizzo, Kanwarpal S Kahlon

**Affiliations:** 1 Rheumatology, University of California Los Angeles David Geffen School of Medicine, Los Angeles, USA; 2 Pathology and Labratory Medicine, University of California Los Angeles David Geffen School of Medicine, Los Angeles, USA; 3 Pathology and Laboratory Medicine, University of California Los Angeles David Geffen School of Medicine, Los Angeles, USA; 4 Hematology and Oncology, University of California Los Angeles David Geffen School of Medicine, Los Angeles, USA

**Keywords:** cytopenias, marginal zone lymphoma, non-hodgkin lymphoma, primary sjögren's syndrome, rituximab, splenomegaly

## Abstract

Primary Sjögren’s syndrome (pSS) is associated with an increased risk of lymphoproliferative disorders, including non-Hodgkin lymphoma (NHL). We report the vase of a woman with longstanding pSS who presented with progressive weight loss, massive splenomegaly, and cytopenias. Extensive hepatologic evaluation, including transjugular liver biopsy, showed no evidence of cirrhosis or intrinsic liver disease. Bone marrow biopsy established the diagnosis of a low-grade B-cell lymphoma consistent with marginal zone lymphoma. The patient was treated with rituximab monotherapy and demonstrated a good clinical response.

This case underscores the importance of maintaining a high index of suspicion for lymphoproliferative disease in pSS patients who develop unexplained splenomegaly and cytopenias, even in the absence of lymphadenopathy or classic B symptoms. When hematologic abnormalities persist despite negative preliminary investigations, bone marrow biopsy remains essential for timely and definitive diagnosis.

## Introduction

Primary Sjögren’s syndrome (pSS) is a chronic systemic autoimmune disease characterized by lymphocytic infiltration of exocrine glands, leading to sicca symptoms and a wide spectrum of extraglandular manifestations [1]. Among autoimmune conditions, pSS carries the highest risk of non-Hodgkin lymphoma (NHL), with relative risk estimates ranging from 13.76 to 44 times that of the general population [2,3]. The lifetime risk is approximately 5%-15%, with mucosa-associated lymphoid tissue (MALT) lymphomas being most common, followed by diffuse large B-cell lymphomas and nodal marginal zone lymphomas [4].

Lymphomagenesis in pSS is thought to result from chronic antigenic stimulation and persistent B-cell activation, progressing from polyclonal expansion to monoclonal proliferation and, ultimately, malignant transformation [4]. Clinical predictors of lymphoma include persistent salivary gland enlargement, splenomegaly, lymphadenopathy, palpable purpura, peripheral neuropathy, and cytopenias, while laboratory predictors include hypocomplementemia (particularly low C4), cryoglobulinemia, monoclonal gammopathy, and elevated beta-2-microglobulin [5,6].

Although splenomegaly is a known risk factor for lymphoma in pSS, massive splenomegaly with cytopenias as the leading presentation is uncommon. We report a case of low-grade B-cell lymphoma diagnosed on bone marrow biopsy in a patient with established pSS who presented with progressive hepatosplenomegaly and cytopenias, highlighting the diagnostic challenges and the need for thorough hematologic evaluation in this clinical context.

## Case presentation

A 65-year-old woman with a five-year history of pSS presented with progressive abdominal discomfort, weight loss, and fatigue. She was diagnosed with pSS in March 2020 based on positive serology (ANA 1:80, anti-SSA/Ro antibodies), sicca symptoms, and a positive minor salivary gland biopsy. Her disease had remained clinically stable on hydroxychloroquine 200 mg daily, although she consistently exhibited low complement levels. Past medical history included oophorectomy, hysterectomy, and cholecystectomy.

In March 2025, she began experiencing postprandial abdominal discomfort. By May 2025, she presented to the emergency department with worsening abdominal pain, nausea, and vomiting. She subsequently developed progressive weight loss from 135 to 111 pounds over three months, with appetite suppression attributed to splenic compression and malabsorption symptoms requiring digestive enzyme supplementation.

Physical examination revealed hepatomegaly with the spleen tip palpable approximately 10-12 cm below the left costal margin, consistent with massive splenomegaly. No lymphadenopathy, skin rashes, purpura, or vasculitic lesions were observed. Neurological examination was unremarkable.

Laboratory studies demonstrated progressive cytopenias. In August 2025, hemoglobin ranged from 10.8 to 11.2 g/dL (reference: 11.6-15.2 g/dL), white blood cell count from 2.83 to 3.09 × 10³/μL (reference: 4.16-9.95 × 10³/μL), and platelet count from 108 to 109 × 10³/μL (reference: 143-398 × 10³/μL). Sjögren's disease markers showed ESR and CRP within normal limits, C3 of 4 mg/dL (chronically low), normal C4, normal LDH, and beta-2-microglobulin of 3.7 mg/L (chronically elevated, reference <2.4 mg/L). Liver-kidney microsomal, anti-mitochondrial, and smooth muscle antibodies were negative (Table 1).

**Table 1:** Key laboratory values ANA: antinuclear antibody; dsDNA Ab: anti-double-stranded DNA antibodies; EIA: enzyme immunoassay, IFA: immunofluorescence assay; SSA: anti–Sjögren's-syndrome-related antigen A autoantibodies; SSB: anti–Sjögren's-syndrome-related antigen B autoantibodies; B2: beta-2; LD: lactate dehydrogenase; C3: complement component 3; C4: complement component 4; IgG: immunoglobulin G; IgA: immunoglobulin A

Name	Interpretation	Value	Normal Reference Range
ANA Titer	High	1:80	<1:40 titer
ANA Pattern	—	Speckled	—
dsDNA Ab (EIA)	Normal	<=200	<=200
dsDNA Ab (IFA)	High	1:80	<1.10
SSA	High	110	<20 U
SSB	Normal	<20	<20 U
B2-Microglobulin	High	3.3	0.8-2.4 mg/L
Cryoglobulin	Normal	Negative 72 Hour	Negative 72 Hour
LD	Normal	167	125-156 U/L
C3	Normal	95	86-175 mg/dL
C4	Low	6	10-40 mg/dL
Erythrocyte Sedimentation Rate	Normal	9	<=25 mm/hr
C-Reactive Protein	Normal	0.3	<0.8
IgG	Normal	864	700-1600 mg/dL
IgA	Low	37	76-426 mg/dL
Alpha 1 Globulins %	High	5.8	2.9-4.9 %
Alpha 1 Globulins	High	0.4	0.2-0.3 g/dL
White Blood Cell Count	Low	3.09	4.16-9.95 x10E3/uL
Hemoglobin	Low	10.8	11.6-15.2 g/dL
Hematocrit	Low	34.1	34.9-45.2 %
Red Blood Cell Count	Low	3.91	3.96-5.09 x10E6/uL
Platelet Count	Low	108	143-398 x10E3/uL
Absolute Lymphocyte Count	Low	0.47	1.30-3.40 x10E3/uL
Urine Protein/Creatinine Ratio	Normal	0.1	0.0-0.4
Serum Immunofixation	Normal	No monoclonal immunoglobulins present	—

**Figure 1:**
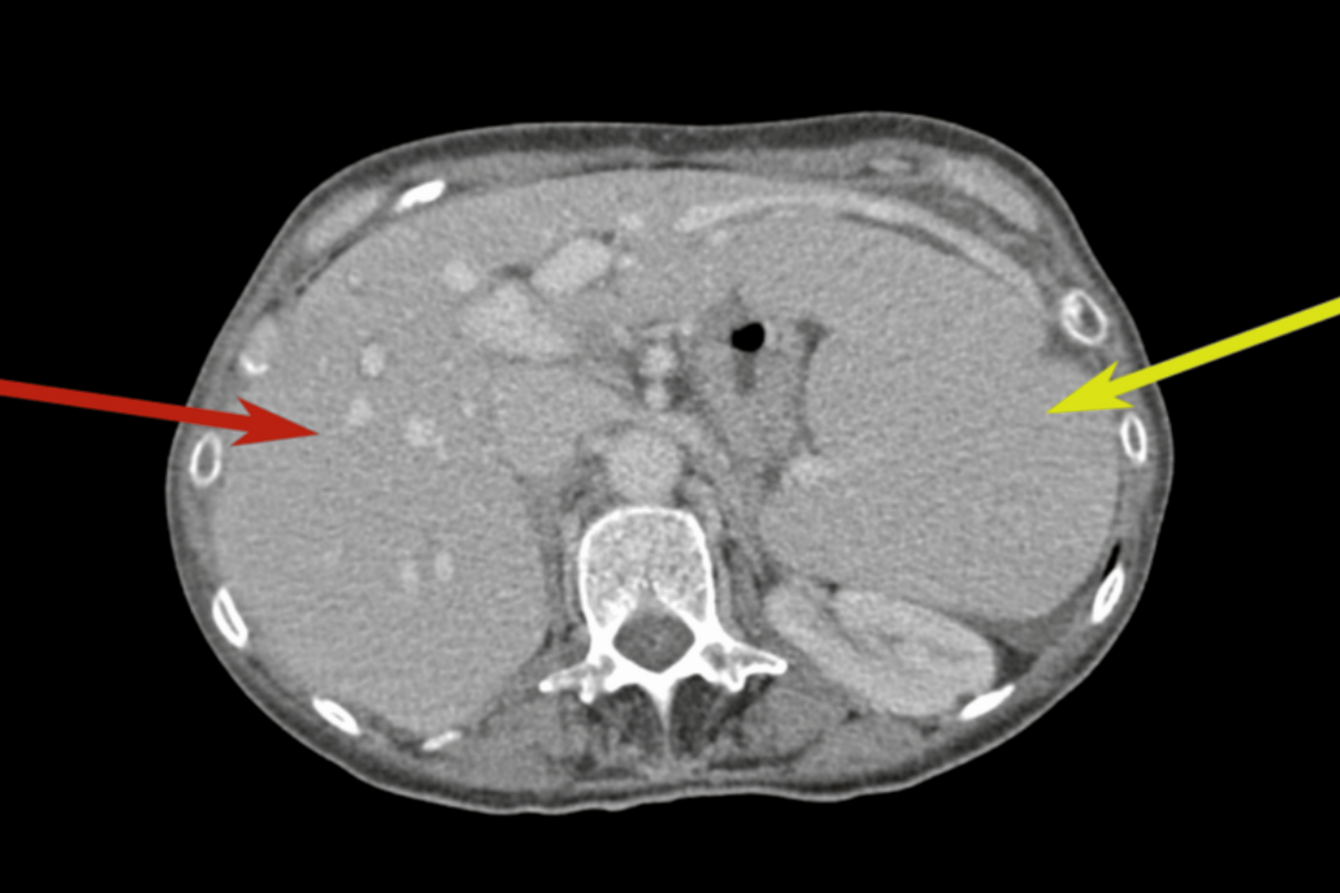
CT scan demonstrating hepatosplenomegaly The red arrow indicates hepatomegaly and the yellow arrow highlights splenomegaly.

**Figure 2:**
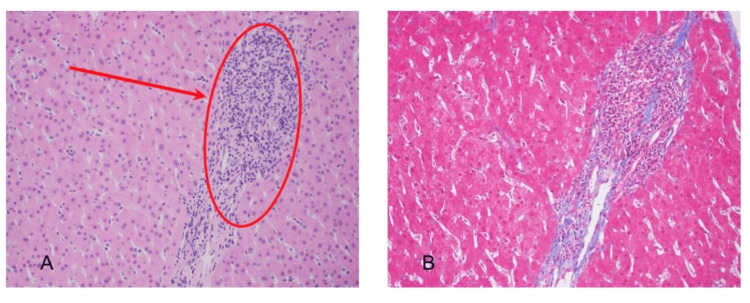
Liver with non-specific periportal inflammation (A) Photomicrograph of liver parenchyma and a portal tract containing mild periportal inflammation (red oval and arrow) without interface or lobular activity; (B) Masson trichrome stain does not reveal periportal fibrosis.

Magnetic resonance imaging and elastography showed no evidence of cirrhosis. Given hepatosplenomegaly with portal hypertension, the patient was referred to hepatology in May 2025. CT imaging revealed hepatosplenomegaly with marked splenic enlargement and a dilated portal vein suggestive of portal hypertension (Figure 1). Transjugular liver biopsy (Figure 2) demonstrated normal hepatic architecture with healthy parenchyma and normal hepatic veins, excluding cirrhosis, fibrosis, or infiltrative disease. Idiopathic portal hypertension, a known association with Sjögren's syndrome, was considered.

**Figure 3:**
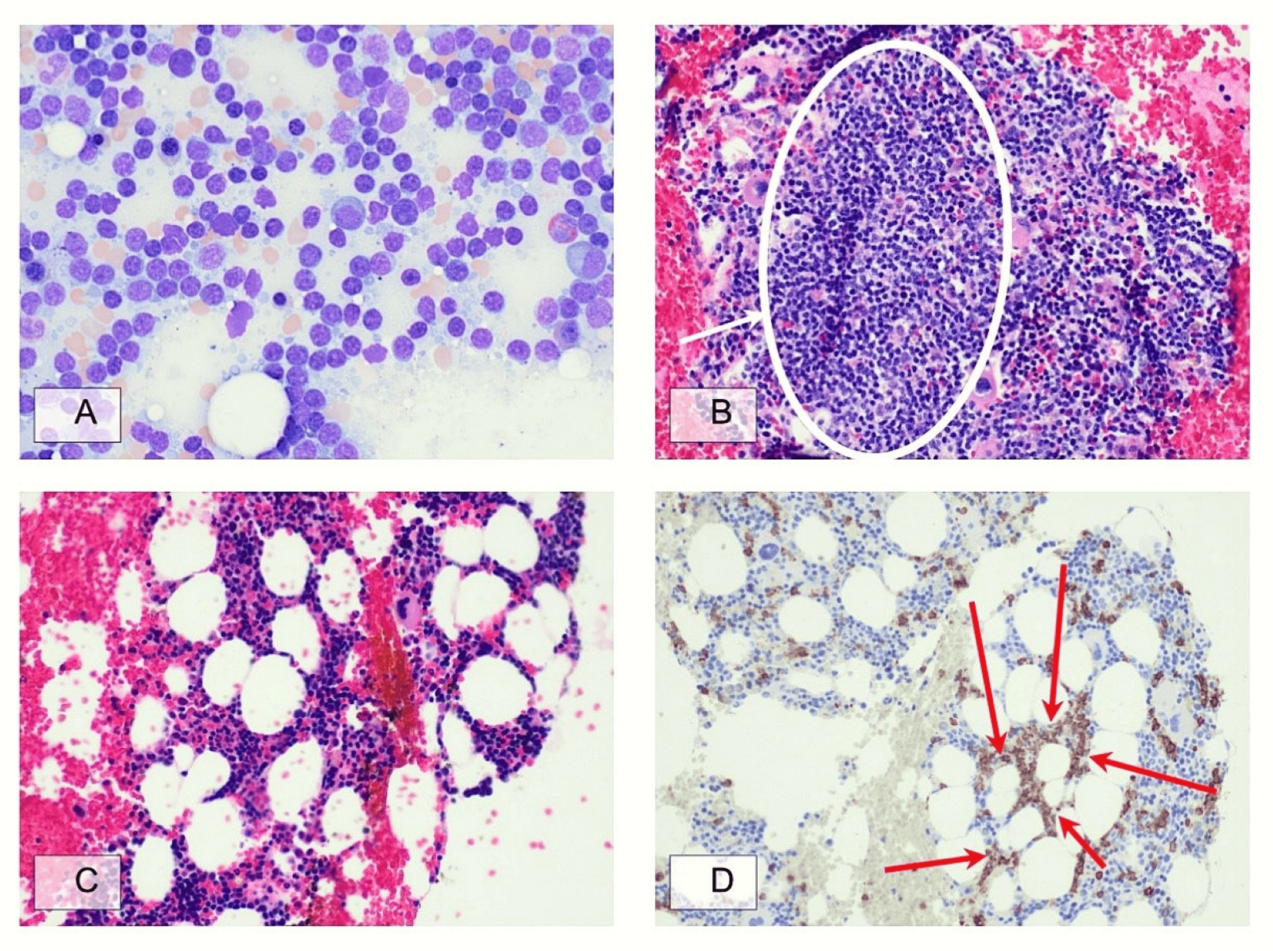
Bone marrow involvement by a B-cell lymphoproliferative disorder (A) Bone marrow aspirate smear displaying a relative increase in the proportion of mature-appearing lymphocytes; (B) Bone marrow clot section with prominent lymphoid aggregate (white oval); (C) Bone marrow core section showing interspersed small lymphocytes in a background of trilineage hematopoiesis; (D) CD20 immunohistochemistry highlighting mildly increased atypical B lymphocytes in cords and small aggregates (red arrows).

**Figure 4:**
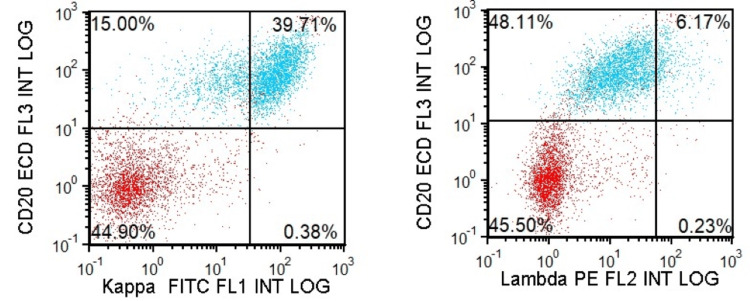
Flow cytometry showing a monotypic kappa light chain restricted B-cell population Lymphocyte-gated (high CD45 expression and low side scatter) population contains a subset of CD20-positive lymphocytes with kappa light chain restriction.

Due to progressive splenomegaly, persistent cytopenias, and weight loss despite negative hepatic evaluation, the patient was referred to hematology. A bone marrow biopsy in September 2025 (Figure 3) revealed normocellular marrow with trilineage hematopoiesis and focal lymphoid aggregates involving approximately 10-15% of marrow cellularity. Flow cytometry (Figure 4) detected a monotypic kappa-restricted B-cell population comprising 11% of total cells with a CD5-negative and CD10-negative immunophenotype. Iron stains showed adequate stores. Cytogenetics revealed a normal karyotype (46,XX). Fluorescence in situ hybridization (FISH) was negative for common lymphoma-associated translocations. Molecular analysis of the bone marrow specimen revealed variants in FAS, SPEN, and ZMYM3, with no MYD88 or BRAF mutations detected. Additionally, a NOTCH2 variant at a very low variant allele frequency was also identified. Alterations in these genes have been reported in various B-lymphoproliferative disorders, such as splenic B-cell lymphomas. The pathologic diagnosis was consistent with a low-grade B-cell lymphoproliferative disorder.

Based on bone marrow findings and symptomatic presentation, treatment with rituximab monotherapy was initiated. The patient received her first dose of rituximab 375 mg/m² on September 26, 2025, and was scheduled to receive weekly infusions for a total of four treatments. She completed all four weekly doses without complications. At follow-up on November 17, 2025, she reported no adverse effects with stable weight at 123 pounds. On examination, the liver and spleen were no longer palpable. She continued hydroxychloroquine 200 mg daily for underlying Sjögren's syndrome.

## Discussion

This case illustrates important considerations in managing pSS patients who develop lymphoproliferative complications. Our patient presented with an unusual manifestation of low-grade B-cell lymphoma, massive splenomegaly with portal hypertension mimicking primary hepatic disease, occurring without significant lymphadenopathy and with no constitutional B symptoms except weight loss.

Patients with pSS have substantially elevated NHL risk, with meta-analyses demonstrating a 13.76-fold increased risk compared to the general population [2]. A recent meta-analysis found that pSS was associated with an increased risk of hematological malignancy with a pooled standardized incidence ratio (SIR) of 11.55, including NHL (pooled SIR 13.71) and other hematological malignancies [7]. The predominant histologic subtypes are B-cell lymphomas, particularly MALT lymphomas (approximately 65% of cases), followed by nodal marginal zone and diffuse large B-cell lymphomas [4]. The pathogenesis involves chronic B-cell activation driven by persistent antigenic stimulation, evolving through polyclonal expansion, oligoclonal/monoclonal predominance, and malignant transformation [4].

Multiple predictors of lymphoma development have been identified. Clinical predictors include persistent salivary gland enlargement, splenomegaly, lymphadenopathy, palpable purpura, and peripheral neuropathy. Laboratory predictors include hypocomplementemia (particularly low C4 and C3), cryoglobulinemia, cytopenias, monoclonal gammopathy, and elevated beta-2-microglobulin [5,6]. Our patient exhibited several high-risk features: chronic hypocomplementemia, elevated beta-2-microglobulin, progressive pancytopenia, splenomegaly, and anti-SSA positivity. Prediction models have shown that patients with three to six risk factors have a 39.9% probability of NHL development [8].

The presentation with massive splenomegaly and portal hypertension created diagnostic complexity. Idiopathic portal hypertension is a recognized but uncommon association with pSS, likely related to lymphocytic infiltration of portal vessels or immune-mediated endothelial injury [9]. This can present with hepatosplenomegaly and portal vein dilation without cirrhosis. Similar diagnostic challenges have been reported by other investigators. Zhang et al. (2024) described a 49-year-old male patient with splenic marginal zone lymphoma presenting with splenomegaly and cytopenias who was successfully treated with rituximab-based chemotherapy [10]. In a case series from China, investigators reported two patients with pSS and massive splenomegaly who underwent splenectomy to exclude lymphoma and improve cytopenias, highlighting splenomegaly as a rare but clinically significant finding in pSS when complicated by lymphoma [11].

The extensive hepatology workup in our case, including transjugular liver biopsy, effectively excluded cirrhosis and primary hepatic pathology, redirecting focus toward hematologic causes. The persistence of cytopenias despite negative hepatic findings, combined with high-risk pSS features, prompted bone marrow evaluation that yielded the diagnosis. This systematic approach is critical given the overlap between idiopathic portal hypertension and lymphoproliferative disorders in pSS.

The bone marrow biopsy revealed a low-level involvement by a B-cell lymphoproliferative disorder, such as a low-grade mature B-cell lymphoma. The immunophenotypic profile (CD5-negative, CD10-negative, kappa-restricted) excluded chronic lymphocytic leukemia/small lymphocytic lymphoma (typically CD5-positive) and follicular lymphoma (typically CD10-positive). The absence of MYD88 excluded lymphoplasmacytic lymphoma, and the absence of a BRAF mutation excluded hairy cell leukemia. These results, together with the molecular profile, the relatively low marrow burden (10%-15% involvement), and the clinical features suggesting primarily splenic disease, overall favor a diagnosis of splenic marginal zone lymphoma.

In a large harmonized dataset study (2023) analyzing 878 pSS patients, investigators found that 16.7% developed NHL, with MALT lymphoma being the most common (76%), followed by diffuse large B-cell lymphoma (9%) and nodal marginal zone lymphoma (6.6%) [12]. Notably, in the nodal marginal zone lymphoma subset, splenomegaly was found in 75% of patients (6/8), consistent with our case presentation [12]. This finding supports that splenomegaly is a cardinal feature of marginal zone lymphoma in pSS patients.

Rituximab, a chimeric anti-CD20 monoclonal antibody, has become an established treatment for both pSS and pSS-associated lymphomas. While large randomized trials of rituximab for pSS disease activity have shown mixed results for sicca symptoms [13], rituximab has demonstrated efficacy in treating systemic manifestations and B-cell lymphoproliferation. In a retrospective study of 35 pSS patients with MALT lymphoma, rituximab monotherapy achieved a complete response in multiple patients, with particularly good outcomes in those with localized disease [14]. The study noted that patients with initially high SS disease activity required more aggressive treatment approaches [14].

In our patient, rituximab monotherapy (375 mg/m² weekly × 4) was appropriate given the low-grade histology and symptomatic splenomegaly with cytopenias. This approach allows for B-cell depletion, potentially addressing both the lymphoproliferative process and underlying pSS disease activity. The patient tolerated initial treatment well, and recent data support its safety: a 2025 meta-analysis demonstrated that rituximab can significantly improve disease activity scores in pSS patients, though effects on glandular function remain limited [15]. Importantly, adverse events were comparable to control groups, supporting the safety profile of rituximab in this population [15].

The prognosis for low-grade B-cell lymphomas complicating pSS is generally favorable, particularly for MALT-type lymphomas characterized by indolent behavior. However, transformation to high-grade lymphoma can occur and warrants long-term surveillance, including monitoring of disease activity markers, surveillance imaging, and vigilance for transformation.

This case reinforces several principles. First, splenomegaly and cytopenias in pSS patients warrant thorough hematologic investigation, even when other explanations are being considered. Second, when cytopenias persist and lymphoproliferative disorder is suspected, bone marrow biopsy provides a definitive diagnosis. Third, multidisciplinary collaboration is essential to systematically exclude other diagnoses. Fourth, regular assessment of lymphoma risk factors allows for appropriate surveillance and early detection. Finally, rituximab represents an effective treatment option for pSS-associated low-grade B-cell lymphomas.

## Conclusions

We present a case of low-grade B-cell lymphoma in a patient with pSS, highlighted by massive splenomegaly, portal hypertension, and cytopenias. The patient showed clear clinical improvement with rituximab monotherapy, including weight gain and resolution of palpable splenomegaly. This case underscores the diagnostic challenges of atypical lymphoproliferative presentations in pSS and reinforces the need for high clinical suspicion, timely bone marrow evaluation, and ongoing surveillance for progression or transformation.
